# A modified triangular flap suture method used for inferior third molar extraction: A three-arm randomized clinical trial for the assessment of quality of life

**DOI:** 10.4317/medoral.25859

**Published:** 2023-06-18

**Authors:** Tong-Yue Wang, Zhi-Ping Wang, Zhao-Qiang Zhang, Xiang-Huai Zheng, Yuan Du, Jin-Yuan Guo

**Affiliations:** 1 Center of Oral Implantology, Stomatological Hospital, School of Stomatology, Southern Medical University, Guangzhou, China; 2 Department of Oral and Maxillofacial Surgery, Stomatological Hospital, School of Stomatology, Southern Medical University, Guangzhou, China; 3 Department of Stomatological department, Guangdong second provincial general hospital, Guangzhou, China

## Abstract

**Background:**

The purpose of this study is to explore whether decreasing the number of sutures can improve the quality of life after inferior third molar extraction.

**Material and Methods:**

This study used a three-arm randomized design that included 90 individuals. Patients were randomized and divided into three groups—the airtight suture group (traditional), the buccal drainage group, and the no-suture group. Postoperative measurements, including treatment time, visual analog scale, questionnaire on postoperative patient quality of life, and details about trismus, swelling, dry socket, and other postoperative complications were obtained twice and the mean values were recorded. To verify the normal distribution of the data, the Shapiro-Wilk test was performed. The statistical differences were evaluated using the one-way ANOVA and the Kruskal-Wallis test with Bonferroni post-hoc correction.

**Results:**

The buccal drainage group showed a significant decrease in postoperative pain and better speech ability than the no-suture group on the 3st day, with a mean of 1.3 and 0.7 (*P* < 0.05). The airtight suture group also showed similar eating and speech ability, which was better than the no-suture group, with a mean of 0.6 and 0.7 (*P* < 0.05). However, no significant improvements were noted on the 1st and 7th days. The surgical treatment time, postoperative social isolation, sleep impairment, physical appearance, trismus, and swelling showed no statistical difference between the three groups at all measured times (*P* > 0.05).

**Conclusions:**

Based on the above findings, the triangular flap without a buccal suture may be superior to the traditional group and no-suture group in less pain, and better postoperative patient satisfaction in the first 3 days and may be a simple and viable option in clinical practice.

** Key words:**Buccal suture, inferior third molars, quality of life, triangular flap.

## Introduction

Impacted third molars are most commonly found among wisdom teeth and lead to clinical diseases, including pericoronitis, adjacent teeth damage, and dentigerous cyst. However, many patients have fears about tooth extraction due to postoperative pain, swelling, trismus, and nerve injury that severely affects the quality of life (1). Therefore, clinicians use various surgical approaches to reduce the severity of postoperative complications; apart from using painkillers and dexamethasone, different flap designs and suturing technologies play important roles in affecting the frequency and severity of postoperative complications (2,3).

Among all the types of flap designs for the removal of an impacted mandibular third molar, the most widely clinically used flap design is the triangular flap (4-6). As a primary closure, the incision of a traditional triangle flap is large, and the socket is sealed using the airtight mucosal flap ensuring no contact with the oral cavity, which may lead to severe trismus and pain (7). Dubois *et al*. first suggested that the procedure of choice after removal of an impacted mandibular third molar was a secondary closure that facilitates the drainage of the inflammatory exudate, leading to less postoperative pain and edema, thus, enhancing patient comfort (8). Pachipulusu *et al*. stated that a secondary closure was better than a primary closure in terms of postoperative pain, swelling, and trismus (9). However, an open third molar socket exposed to the oral cavity results in food accumulation and results in a protracted healing period that increases the risk of wound dehiscence and postoperative infection.

In some cases, the buccal drainage method (without a buccal suture) or the no-suture method are used to release postoperative pain and edema. However, through a literature review, we determined there is still no consensus on whether these two secondary closure methods could improve the patient’s quality of life after a third molar extraction. Thus, the need for suturing in some situations needs to be fully considered. Therefore, in this study we aim to investigate if these two suture methods are more advantageous than the traditional method.

## Material and Methods

- Study design and sample

This randomized, prospective, three-arm clinical study consisted of patients who presented to the Department of Oral and Maxillofacial Surgery for surgical removal of a bilaterally impacted mandibular third molar from October 2021 to April 2022. Patients participated voluntarily and signed an informed consent form as well as understood and complied with the research scheme. The study protocol was reviewed and approved by the Ethics Committee of the Stomatological Hospital, Southern Medical University, Guangzhou. All procedures performed in studies involving human participants were in accordance with the 1964 Declaration of Helsinki and its later amendments or comparable ethical standards. The objective of the procedure was explained during the first appointment. All the patients were informed of the potential complications and benefits. All patients were treated by the same surgeon (G.J.Y), a specialist with more than 10 years of experience in oral and maxillofacial surgery. The reporting of the methodology used in this study conforms with the CONSORT Statement (10).

- Inclusion and exclusion criteria

The Winter and Pell and Gregory classifications were used to evaluate the inclusion and exclusion criteria. The molars were horizontally/mesio-angularly impacted, and all teeth were partially or completely covered by mucosa. The region, size, and resistance distribution of the impacted molars were similar as seen on panoramic radiographs. Patients with any systemic disease, poor oral hygiene, age < 18, or who failed to attend follow-ups were excluded from this study. Smokers were not excluded from this study, but all the patients were informed not smoke at least two weeks after surgery. Patients with differences in operation time that exceeded 5 minutes were also excluded from the study.

- Sample size calculation

A priori power analysis was carried out during the planning stage of this experiment to determine the ideal sample size. The ideal sample size was calculated using G* power software version 3.1.9.7 to ensure adequate computing power for the study. To detect a difference between the groups with a two-sided 5% significance level and a power of 80%, a sample size of 84 patients was necessary, and this was increased to 90 patients to compensate for possible losses.

- Randomization

**Figure 1:**
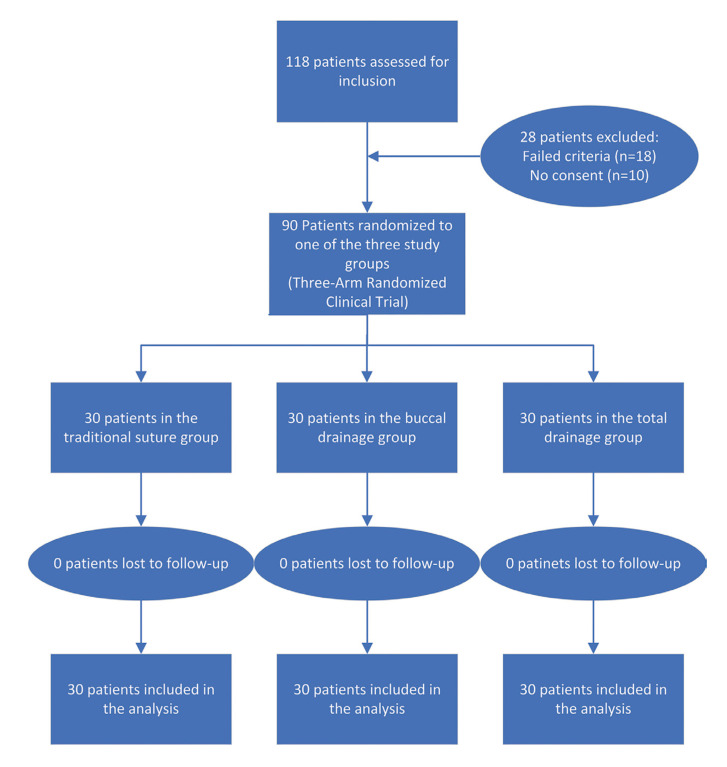
CONSORT flow diagram of the patients in the trial.

A block permuted randomisation was used with variable block size (3-6-9). The block size was not disclosed, to ensure concealment. The randomization list was excel-generated to decide which suture method to use by an independent statistician. Patients were randomized and divided into three groups (1:1:1)—the traditional airtight suture method group, the buccal drainage method group, and the no-suture method group. The result was concealed using sequentially numbered, opaque, sealed envelopes, and kept by a nurse who was not involved in this study. Allocation concealment was intended to prevent selection bias and to protect the assignment sequence until the first procedure. The nurse then informed the attending doctor of the method to use before the operation (Fig. 1).

- Surgical procedure

**Figure 2:**
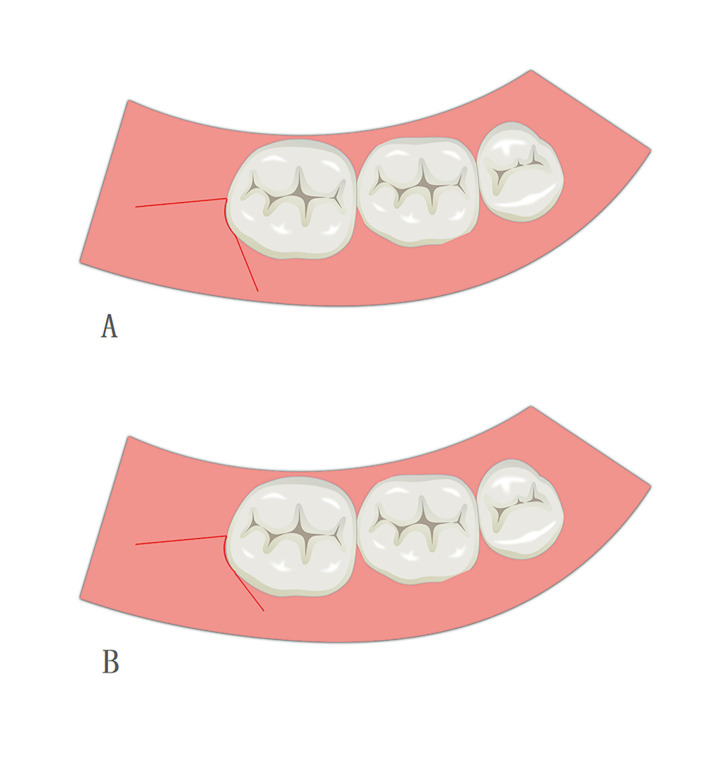
Incision line. (a) Incision line used in this study. (b) Traditional incision line.

**Figure 3:**
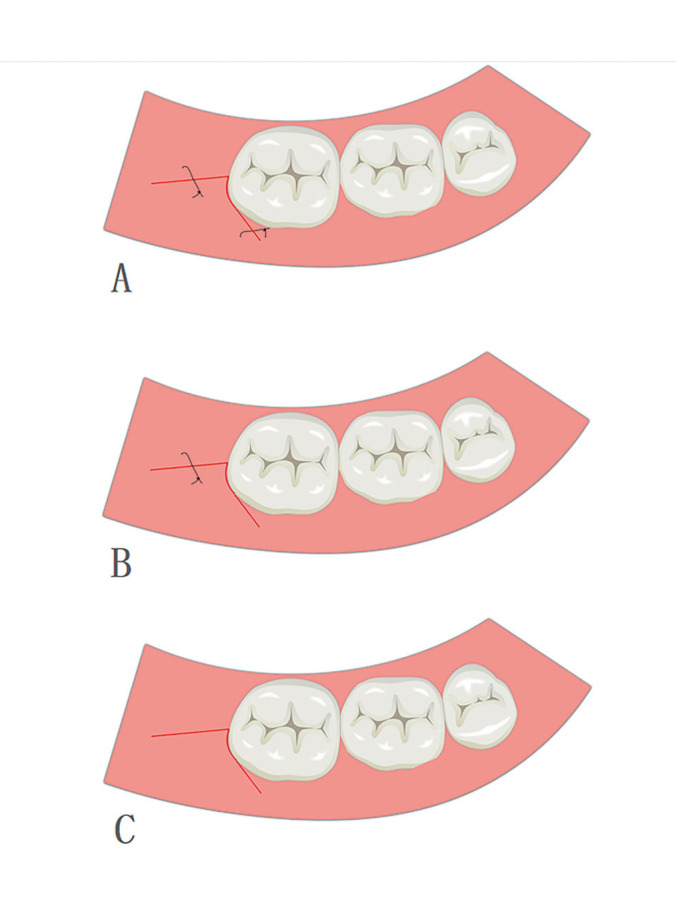
Suture method in this study. (a) Airtight suture method. (b) Buccal drainage method. (c) No-suture method.

The surgery was carried out with the patients under local anesthesia. The anesthetic used was Articaine in a 4% solution with additional epinephrine in a concentration of 1:100000 (Primacaine, France). All patients were cut with an electric knife (XO Odontosurge4, Denmark). The traditional triangular flap incision was implemented as described by Szmyd *et al*. (11). The distal incision started from the middle point of the second molar’s distal gingival margin and extended postero-laterally. The mesio-buccal incision started from the distobuccal axial angles of the adjacent tooth and was at a 45° angle to the gingival margin. The incision moved downward and forward but did not surpass the bottom of the transitional channel. In this study, in order to use suture-less releasing incision to reduce complexity and operation time. The vertical incision was closer to the gingival margin to make the vertical incision cover all the bone defect area. Second, the incision should be as short as possible, so there in no need for suturing (Fig. 2). After flap surgery, the incision did not deviate to the lingual side and was long enough to expose the buccal and distal bone surfaces. The high-speed contra-angle handpiece was used to divide the horizontally impacted wisdom teeth into the crown and root allowing for the separate removal of the teeth. All sutures were interrupted suture with non-absorbable silk (Mersilk, China). In the airtight suture method group, one stitch was placed in the mesio-buccal incision and another stitch in the distal incision. In the buccal drainage method group, the distal incision was firmly stitched with one stitch with no-sutures on the buccal side. In the no-suture method group, no-sutures were used for closure (Fig. 3). The patients were kept in the hospital under observation for 30 minutes, and then rechecked for flap position and hemorrhage. If there is still some bleeding, the patients will bite a new gauze until the hemostatic effect is achieved.

- Postoperative Medicaltions

All patients in the study routinely received amoxicillin (oral 500 mg every 8 hours) for 3 days after surgery. Chlorhexidine solution (0.12%) was administered 3 times a day for 7 days. Ibuprofen was taken only if required to control post-operative pain and edema.

- Postoperative follow-up and measurement

All patient treatment times were recorded (from the first incision to the last suture);the operator and the dental assistant who performed the surgical interventions were not blinded due to the nature of the interventions. One author, who performed all the measurements and was responsible for the calculations and calibrations, was not involved in the selection and intervention of the participants. Postoperative measurements, that included details of pain, trismus, swelling, quality of life, dry socket, and other postoperative complications, were repeated twice and the mean values were recorded. The data collection methods are as follows:

All patients were given a 10-cm standard visual analog scale (VAS) form—the scoring ranged from “0” meaning no pain on the left to “10” meaning the worst possible pain on the right. Patients were asked to indicate the point on the scale that best corresponds to their pain on postoperative days 1, 3, and 7. They were also asked to record analgesic requirements for the 7 days after surgery. Trismus was performed by measuring the distance between the mesial-incisal corners of the upper and lower right central incisors at a maximum opening of the jaws for pre-operative and post-operative (after 1, 3, and 7 days) conditions. Facial swelling was determined based on soft tape measurements between the tragus and the soft tissue pogonion, the tragus and the lateral corner of the mouth, the lateral corner of the eye, and the angle of the mandible. Percentages were calculated from the differences between the pre-operative and the 1, 3, and 7 days post-operative measurements. The results were then divided by the value obtained in the pre-operative period and multiplied by 100, as described in Amin *et al*. (12).

Another important measurement was the modified questionnaire on postoperative patient quality of life (QOL) (13-15). The questionnaire involved different items addressing social and working isolation, eating ability, speaking ability, sleep impairment, and physical appearance. The patients received the questionnaire to be completed post-surgery on days 1, 3, and 7, and were returned during the suture removal on day 7. They were instructed to answer the questions and rate them on a 4-point scale (never to very much) concerning their experience with the third molar surgery.

- Statistical analysis

Statistical analysis was performed using the SPSS for Windows version 23.0 (SPSS Inc., Chicago, IL). The Shapiro-Wilk test was performed, and the results were expressed as mean ± standard deviation in order to verify the normal distribution of the data. The statistical differences were performed using the Pearson's chi-squared test, one-way ANOVA, and the Kruskal-Wallis test with Bonferroni post-hoc correction, for data normally distributed and non-normally distributed,. The level of significance was set at *P* value < 0.017 for all tests.

## Results

Demographic characteristics are described in Table 1. In total, 90 individuals (36 males and 54 females; mean age 26.2 years; age range 19-39 years) were included in this study. It has been demonstrated that operating time is a reliable measure of surgical difficulty within mandibular third molar surgeries; in the present study operating time was comparable between the airtight suture method group (9.2 ± 2.3 min), the buccal drainage method group (9.0 ± 2.2 min), and the no-suture method group (8.6 ± 1.9 min) without significant differences (*P* > 0.05). Painkillers were needed in the airtight suture method (2.7 ± 1.7), the buccal drainage method (2.4 ± 1.7), and the no-suture method (3.0 ± 1.6) groups, without significant difference (Table 1).

In the comparison between the three groups on the 1st day after surgery, all values were better in the buccal drainage method group than in the airtight suture method and the no-suture method group, but not significantly. Compared to the first day, the buccal drainage method group showed significant postoperative pain decrease and better speech ability than the no-suture method group on the 3rd day, with a mean of 1.3 and 0.7, respectively (*P* <0.05). The traditional airtight suture method groupalso showed similar eating and speech ability that was better than the no-suture method group, with a mean of 0.6 and 0.7, respectively (*P* < 0.05). However, these differences were not significant on the 7th day. The postoperative social isolation, sleep impairment, physical appearance, trismus, and swelling showed no statistical difference between the three groups at all measured times (*P* > 0.05) (Table 2).

**Figure 4:**
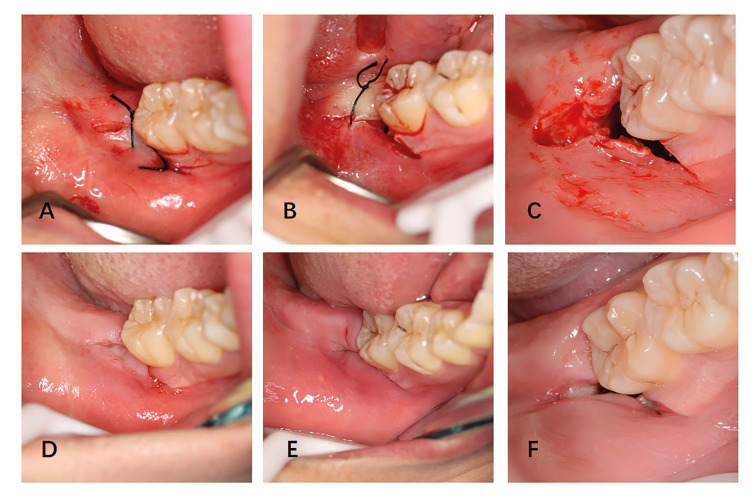
Clinical photograph of the surgical area. (a) Airtight suture method, one stitch in the mesio-buccal incision and another stitch in the distal incision. (b) Buccal drainage method, with only one stitch in the distal incision. (c) No-suture method, with no sutures used for closure. (d) Clinical outcomes of the airtight suture method group after 7 days. (e) Clinical outcomes of the buccal drainage method group after 7 days. (f) Clinical outcomes of the no-suture method group after 7 days.

One case of alveolitis was also found in the no-suture method group after surgery; however, the patient recovered quickly after the appropriate treatment was administered. None of the patients exhibited any complications such as nerve injury and hematoma, and all patients recovered without any complications from the procedure. No patients discontinued the trial or were lost to follow-up (Fig. 4).

**Table 1:**
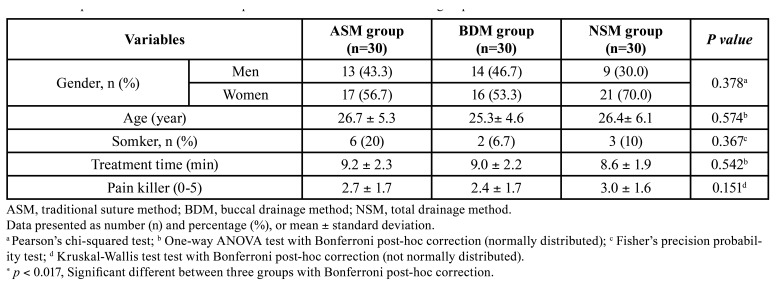
Comparison of treatment time and pain killer difference between three groups.

**Table 2:**
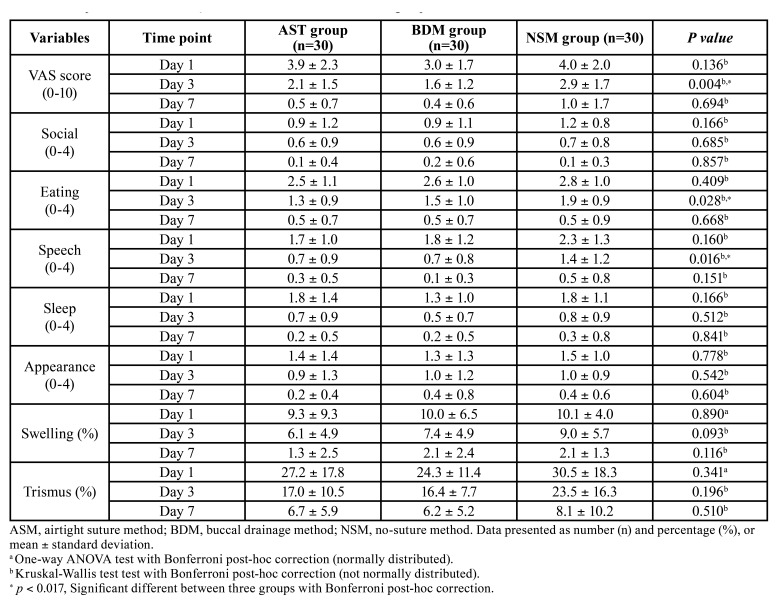
Comparison of VAS and QOL scores difference between three groups.

## Discussion

The alleviation of patient discomfort from pain, swelling, and trismus following an impacted mandibular third molar removal presents a continual challenge to oral surgeons. Thus, there are many kinds of flap designs used while removing an impacted mandibular third molar with the aim of recovering the soft tissues for better healing (2,6,13,16-20). Among all the types of flap design, the triangular flap and envelope flap design are the most widely used for lower third molar surgery. The modified triangular flap may be superior to the envelope flap in terms of wound dehiscence and pain. The wound dehiscence in modified triangular flap (10%) is significantly less than envelope flap (57%) (21). This result ties well with previous studies wherein Şimşek Kaya found better postoperative pain in modified triangular flap [3.7] than envelope flap [4.8] in day one (22). Similarly, in our study, the modified triangular flap showed only 3.9 VAS on the first day after surgery. In addition, many studies (3,7,9,23-27) indicate that the wound should not be sutured too firmly, and a drainage system should be set up between the distal side of the second molar and the mesial side of the first molar, including either partial suture or no-suture in both directions. Dentists believe that creating such a drainage pathway for inflammatory exudates helps to reduce facial swelling, pain, and other postoperative complications. However, there is a risk of delayed secondary healing on the gap with food impaction due to gravity; therefore, patients avoid using the affected side to chew, due to concerns of food entering the extraction wound and causing postoperative infection or dry socket. Furthermore, there is still no consensus concerning whether decreased suturing can improve the patients’ quality of life after a third molar extraction.

In this study, patients were randomized and divided into three groups: the airtight suture group, the buccal drainage group, and the no-suture group. We transferred the drainage channel from the occlusal surface to the buccal surface to decrease food impaction. The primary result of the present study is that the buccal drainage method revealed better postoperative patient satisfaction than the airtight suture method at all measured times. This could be explained by the fact that the acute inflammatory response peaks within 24 hours after the surgery and then decreases gradually (28). After 24 hours, the retention of the exudates is less in the partial suture group than in the case of the airtight suture method because of the buccal drainage pathway. These findings concur with previous studies. Hu *et al*. used a rubber drainage on the buccal side, which was proven effective (23). Balamurugan *et al*. created a buccal mucosal-advancement flap technique (3). The flap was mobilized by freeing the mucosa from the underlying periosteum while increasing the vertical release length without using a suture. They found less pain, swelling, and trismus in patients who had undergone the buccal drainage method. On the other hand, we also found that patients who had undergone the no-suture method demonstrated good results but recovered more slowly. This was similar to the finding of Waite *et al*. who used the no-suture method, left a “V” shaped incision for postoperative drainage, and achieved excellent results and outcomes (24). In our study, the buccal drainage group showed significant postoperative pain decrease and better speech ability than the no-suture group on the 3rd day, with a mean of 1.3 and 0.7, respectively (*P* <0.05). However, this difference was not significant on the 7th day. This is in agreement with the findings of Alkadi *et al*. (27) wherein, when compared to the suture-less closure, the one-suture closure showed better healing during the early post-operative period.

With respect to surgical incision, Shevel *et al*. found that when a large incision was made, postoperative pain and swelling were worse, and the large buccal incision easily caused buccal wound dehiscence (29). Thus, in our study, we used a short anterior releasing incision in order to reduce damage and avoid food impaction. We also made the vertical incision closer to the gingival margin to prevent the incision from exceeding past the vestibular sulcus, thereby improving the poor visual field caused by the short incision. This method also reduced the complexity and operation time. For an experienced surgeon the placement of the buccal suture does not take very long; however, for some younger doctors it is quite the opposite, sometimes causing secondary soft tissue lacerations. In addition, it should be considered that buccal cleaning difficulties often lead to food residue on the buccal suture line causing buccal oral odor and may cause secondary infections as well. As in earlier studies, Nayak *et al*. used a suture-less anterior releasing incision (30), the results showed that the suture-less incision decreased postoperative swelling and edema; however, the periodontal healing was poor when compared to the sutured cases. Moreover, the lower the third molar are impact, the larger bone defect will be. Especially when the wisdom teeth completely covered by bone. In order to make the vertical incision cover all the bone defect after operation, the length of the vertical incision needs to be lengthened, and the possibility of postoperative bleeding will be higher. For this kind of patients, I think the buccal drainage method or airtight suture method will be better.

The primary limitation of this method is the uncertainty of whether the secondary closure would induce wound dehiscence due to one case of alveolitis in the suture-less group after surgery. Dehiscence creates a potential trap for food particles and is an excellent environment for bacterial growth, thus leading to postoperative alveolar osteitis, soft tissue abscesses, long-term discomfort, and additional treatment requirements. Further comparative studies involving larger populations are required to determine the best flap technique for a third molar surgery. In addition, we don’t have the long-term evaluation results due to the patients usually refused to hospital after the wound healed especially COVID-19 's epidemic period. In conclusion, the buccal drainage method may have more advantageous than the traditional method and no-suture method in terms of pain as well as having a better impact on QOL during the early postoperative period. The triangular flap without buccal sutures may be a simple and viable option for clinical applications, especially for some buccal mucosa tension is difficult to pull, such as mouth opening limitation and buccal mucosal fibrosis.
